# Colon Carcinogenesis: The Interplay Between Diet and Gut Microbiota

**DOI:** 10.3389/fcimb.2020.603086

**Published:** 2020-12-08

**Authors:** Yean Leng Loke, Ming Tsuey Chew, Yun Fong Ngeow, Wendy Wan Dee Lim, Suat Cheng Peh

**Affiliations:** ^1^ Centre for Biomedical Physics, School of Healthcare and Medical Sciences, Sunway University, Petaling Jaya, Malaysia; ^2^ Faculty of Medicine and Health Sciences, Universiti Tunku Abdul Rahman, Kajang, Malaysia; ^3^ Centre for Research on Communicable Diseases, Universiti Tunku Abdul Rahman, Kajang, Malaysia; ^4^ Department of Gastroenterology, Sunway Medical Centre, Petaling Jaya, Malaysia; ^5^ Ageing Health and Well-Being Research Centre, Sunway University, Petaling Jaya, Malaysia; ^6^ Department of Medical Sciences, School of Healthcare and Medical Sciences, Sunway University, Petaling Jaya, Malaysia

**Keywords:** colorectal cancer, colon carcinogenesis, diet, gut microbiota, protein, fat, carbohydrate, bacteria interaction

## Abstract

Colorectal cancer (CRC) incidence increases yearly, and is three to four times higher in developed countries compared to developing countries. The well-known risk factors have been attributed to low physical activity, overweight, obesity, dietary consumption including excessive consumption of red processed meats, alcohol, and low dietary fiber content. There is growing evidence of the interplay between diet and gut microbiota in CRC carcinogenesis. Although there appears to be a direct causal role for gut microbes in the development of CRC in some animal models, the link between diet, gut microbes, and colonic carcinogenesis has been established largely as an association rather than as a cause-and-effect relationship. This is especially true for human studies. As essential dietary factors influence CRC risk, the role of proteins, carbohydrates, fat, and their end products are considered as part of the interplay between diet and gut microbiota. The underlying molecular mechanisms of colon carcinogenesis mediated by gut microbiota are also discussed. Human biological responses such as inflammation, oxidative stress, deoxyribonucleic acid (DNA) damage can all influence dysbiosis and consequently CRC carcinogenesis. Dysbiosis could add to CRC risk by shifting the effect of dietary components toward promoting a colonic neoplasm together with interacting with gut microbiota. It follows that dietary intervention and gut microbiota modulation may play a vital role in reducing CRC risk.

## Introduction

Colorectal cancer (CRC) is the third most commonly diagnosed cancer and the second most deadly cancer in the world, with about 1.8 million new cases and almost 881,000 deaths in 2018, comprising 5.8% of all cancer deaths that year (Rawla et al., 2019). The CRC global burden is expected to increase by 60% by 2030, to approximately 2.2 million new cases and 1.1 million deaths per annum (Rawla et al., 2019). CRC incidence is three to four times higher in developed countries than developing countries, reflecting the state of CRC as a marker of socioeconomic development (Bray et al., 2018). The known risk factors are low physical activity; overweight and obesity; dietary habits including excessive consumption of red, processed meats and alcohol, and low dietary fibers (World Cancer Research Fund/American Institute for Cancer Research, 2018). The rapid rise of CRC incidence among individuals younger than 50 years old especially in high-income countries (Araghi et al., 2019; Siegel et al., 2019; Vuik et al., 2019) coupled with the fact that most CRC incidents arise sporadically (Yang and Yu, 2018) further point to the influence of lifestyle factors on CRC development.

Human gut microbiota is dominated by three primary phyla, namely Firmicutes (30–50%), Bacteroidetes (20–40%), and Actinobacteria (1–10%) (Gagnière et al., 2016). Strict anaerobes such as *Bifidobacterium*, *Fusobacterium*, *Bacteroides*, *Eubacterium*, *Peptostreptococcus*, and *Atopobium* are the major groups of bacteria in the human gut, whereas facultative anaerobes such as *Lactobacilli*, *Enterococci*, *Streptococci*, and *Enterobacteriaceae are* present in numbers that are 1,000-fold lower (Davis and Milner, 2009). The gut microbiota changes swiftly in terms of variety and composition during the first year of birth and remains relatively constant upon adulthood. Nevertheless, the composition of the resident microbiota may be altered due to environmental factors, predominantly, by the influence of diet (Chassaing et al., 2017; Jahani-Sherafat et al., 2018; Yang and Yu, 2018). It is increasingly apparent that when dietary components change the gut microbial composition and its diversity, the balance between beneficial and detrimental gut microbiota could be disrupted, and the resulting impaired gut homeostasis can set the stage for cancer development (Davis and Milner, 2009; Yang and Yu, 2018; De Almeida et al., 2019). Indeed, many studies have revealed a consistent link between colorectal carcinogenesis and gut microbiota. *Fusobacterium nucleatum* (Kostic et al., 2013; Tahara et al., 2014; Fukugaiti et al., 2015; Viljoen et al., 2015; Li et al., 2016; Mima et al., 2016; Tunsjø et al., 2019), *Streptococcus gallolyticus* (Abdulamir et al., 2010; Butt et al., 2016; Corredoira et al., 2017; Kumar et al., 2017; Kwong et al., 2018), *Clostridium difficile* (Fukugaiti et al., 2015; Zheng et al., 2017)*, Clostridium septicum* (Corredoira et al., 2017; Kwong et al., 2018), *Enterococcus faecalis* (Zhou et al., 2016; Rezasoltani et al., 2018; Geravand et al., 2019), *Escherichia coli* (Buc et al., 2013; Bonnet et al., 2014; Kohoutova et al., 2014; Dejea et al., 2018), *Peptostreptococcus stomatis* (Zeller et al., 2014; Yu et al., 2017), and *Bacteroides fragilis* (Boleij et al., 2015; Zhou et al., 2016; Purcell et al., 2017; Dejea et al., 2018; Kwong et al., 2018; Haghi et al., 2019) are differentially enriched in the fecal or colonic mucosa samples of CRC patients relative to healthy individuals, or in the CRC patient’s tumor tissue relative to adjacent healthy tissue, wherein in some cases, CRC disease status is associated with the abundance of CRC-associated gut microbiota. CRC risk is also associated with the seroprevalence of *Helicobacter pylori* antibodies (Zhang et al., 2012; Epplein et al., 2013; Teimoorian et al., 2018; Butt et al., 2019; ChangxiChen et al., 2019). **Table 1** summarizes the common gut microbiota associated with CRC risk obtained from CRC patients’ and healthy subjects’ mucosa, blood, and stool. This is not an exhaustive summary.

**Table 1:** Gut microbiota associated with colorectal cancer (CRC) risk.

Bacteria			Sample type	Method of bacterial analysis	Association	References
Phylum	Genus	Species				
Fusobacteria	*Fusobacterium*	–	Rectal mucosa, colorectal tissue	qPCR	Significantly higher in CRA patients than in healthy controls, and in tumor tissue than in adjacent normal tissue	(McCoy et al., 2013)
			Stool sample	16S rRNA pyrosequencing	Significantly higher in CRC patients than in healthy controls	(Wu et al., 2013)
		*nucleatum*	Colorectal tissue, stool sample	qPCR	Significantly higher in CRA and CRC patients than in healthy controls	(Kostic et al., 2013)
			Stool sample	qPCR	Significantly higher in CRC patients than in healthy controls or patients with polyps	(Tunsjø et al., 2019)
			Stool sample	qRT-PCR	Significantly higher in CRA patients than in healthy controls	(Fukugaiti et al., 2015)
			Colonic mucosa	qPCR	Significantly higher in tumor tissue than in adjacent normal tissue	(Tahara et al., 2014; Viljoen et al., 2015)
			Colorectal tissue	qPCR	Higher abundance was associated with significantly higher CRC-specific mortality	(Mima et al., 2016)
			Colorectal tissue	FQ-PCR, FISH	Significantly higher in tumor tissue than in adjacent normal tissue; overabundance was significantly associated with lymph node metastasis	(Li et al., 2016)
Firmicutes	*Streptococcus*	*gallolyticus*	Blood	Blood culture test	Patients with history of *S. gallolyticus* bacteremia had significantly higher CRC risk than those without	(Kwong et al., 2018)
			Blood	Multiplex serology	Antibody response to pilus protein Gallo2039, Gallo2178 and Gallo2179 was significantly associated with CRC risk	(Butt et al., 2016)
			Blood	Blood culture test	62.7% of 204 patients with *S. gallolyticus* bacteremia had concurrent CRN	(Corredoira et al., 2017)
			Colorectal tissue, fecal sample	PCR, ISH	Significantly higher in CRC patients than in healthy controls	(Abdulamir et al., 2010)
			Colorectal tissue	qPCR	Significantly higher in tumor than in adjacent normal tissue	(Kumar et al., 2017)
	*Clostridium*	*difficile*	Fecal sample	qRT-PCR	Significantly higher in CRA patients than in healthy controls	(Fukugaiti et al., 2015)
			Fecal sample	Multiplex RT-PCR	Significantly higher in CRC patients with lymph node metastasis than those without	(Zheng et al., 2017)
		*septicum*	Blood	Blood culture test	45.2% of 42 patients with *C. septicum* bacteremia had concurrent CRN	(Corredoira et al., 2017)
			Blood	Blood culture test	Patients with history of *C. septicum* bacteremia had significantly higher CRC risk than those without	(Kwong et al., 2018)
	*Enterococcus*	*faecalis*	Stool sample	qRT-PCR	Significantly higher in CRC patients than in healthy controls or patients with polyps	(Geravand et al., 2019)
			Stool sample	qRT-PCR	Higher abundance significantly correlated with dysplasia grade	(Rezasoltani et al., 2018)
			Colorectal tissue	qRT-PCR	Significantly higher in tumor tissue than in adjacent normal tissue	(Zhou et al., 2016)
	*Escherichia*	*coli*	Colonic tissue	PCR	*E. coli* of phylogenetic group B2 was significantly higher in CRC patients than in diverticulosis patients	(Buc et al., 2013)
			Colorectal tissue	PCR	*E. coli* of phylogenetic group D was significantly higher in CRC patients than in healthy controls; *E. coli* of phylogenetic group B2 was significantly higher in patients with advanced CRA than in patients with non-advanced CRA	(Kohoutova et al., 2014)
			Colonic tissue	PCR	Significantly higher in colon cancer tumors than in diverticulosis mucosa and adjacent normal tissue, and in colon cancer patients of stages III/IV than those of stage I	(Bonnet et al., 2014)
			Colonic mucosa	FISH	Significantly higher pks+ *E. coli* in FAP patients than in healthy controls	(Dejea et al., 2018)
	*Peptostreptococcus*	*stomatis*	Stool sample	16S rRNA sequencing	Significantly higher in CRC patients than in healthy controls	(Zeller et al., 2014)
			Stool sample	qPCR	Significantly higher in CRC patients than in healthy controls	(Yu et al., 2017)
	*Helicobacter*	*pylori*	Blood	ELISA	Significantly higher seroprevalence of *H. pylori* antibodies in CRC patients than in healthy controls	(Zhang et al., 2012)
			Blood	ELISA	Significantly higher seroprevalence of *H. pylori* antibodies in patients with CRC and adenomatous polyps than in healthy controls	(Teimoorian et al., 2018)
			Blood	Multiplex serology	Seropositivity of antibodies against VacA was associated with over 2-fold increase in colon cancer risk	(Epplein et al., 2013)
			Expelled air	Carbon-13 urea breath test	Significantly higher prevalence of *H. pylori* infection in CAP patients than in healthy controls	(ChangxiChen et al., 2019)
			Blood	Multiplex serology	Seropositivity of antibodies against VacA was associated with 11% increase in CRC risk	(Butt et al., 2019)
Bacteroidetes	*Bacteroides*	–	Stool sample	16S rRNA pyrosequencing	Prevalence positively correlated with CRC disease status (based on TNM classification)	(Wu et al., 2013)
		*fragilis*	Stool sample	PCR	Significantly higher in CRC patients than in healthy controls	(Haghi et al., 2019)
			Blood	Blood culture test	Patients with history of B*. fragilis* bacteremia had significantly higher CRC risk than those without	(Kwong et al., 2018)
			Colorectal tissue	qRT-PCR	Significant higher in tumor tissue than in adjacent normal tissue	(Zhou et al., 2016)
			Colonic mucosa	Touchdown PCR	Significantly higher expression of bft gene in stages III/IV CRC patients than in healthy controls	(Boleij et al., 2015)
			Colonic mucosa	qPCR	Presence was significantly associated with low-grade dysplasia, tubular adenomas and serrated polyps	(Purcell et al., 2017)
			Colonic mucosa	FISH	Significantly higher in FAP patients than in healthy controls	(Dejea et al., 2018)

CRC, colorectal cancer; CRA, colorectal adenoma; CRN, colorectal neoplasm*; CAP, colorectal adenomatous polyps; FAP, familial adenomatous polyposis; PCR, polymerase chain reaction; qPCR, quantitative polymerase chain reaction; RT-PCR, real-time polymerase chain reaction; qRT-PCR, quantitative real-time polymerase chain reaction; FQ-PCR, fluorescent quantitative polymerase chain reaction; ELISA, enzyme-linked immunosorbent assay; ISH, in situ hybridization; FISH, fluorescent in situ hybridization; pks, polyketide synthase; VacA, vacuolating toxin A; bft, Bacteroides fragilis toxin.

*includes invasive carcinomas (malignant cells beyond the muscularis mucosa) and premalignant lesions (non-advanced and advanced adenoma).

In this article, an overview of the dietary components of various food classes that have been implicated in the pathogenesis of CRC, the mediating roles of gut microbiota and the different mechanisms involved in CRC initiation or progression are deliberated.

## The Interactions Between Diet, Gut Microbiota, and Colorectal Cancer

### Protein

#### Red Meat and Processed Meat

Epidemiological studies have linked excessive processed meat, and to a lesser extent red meat, consumption to increased risk of CRC (Cross et al., 2010; Oostindjer et al., 2014; Demeyer et al., 2016). Reports published by the Word Cancer Research Fund in 2007 and 2018 indicated a strong evidence that a consumption of red and processed meat can increase CRC risk (World Cancer Research Fund/American Institute for Cancer Research, 2018). Processed meat has been classified as group I carcinogen by the World Health Organization’s International Agency for Research on Cancer (2015), that is similar to the risk category of cigarettes and alcohol. Red meat was classified as Group 2A carcinogen, dictating its probable carcinogenicity (Bouvard et al., 2015). In a meta-analysis published in 2017, a 16 and 22% increment of CRC risk was reported for every 100 and 50g/day additional intake of red meat and processed meat, respectively (Zhao et al., 2017). Several mechanisms have been purported to expound the disease-promoting effect of red and processed meat.

##### N-Nitroso Compounds

N-Nitroso compounds (NOCs) consist of a nitroso group attached to a nitrogen atom and are formed by the reaction of a nitrite compound with amines or amides. NOCs comprise two major chemical classes, namely nitrosamines and nitrosamides. Humans are exposed to the highly mutagenic NOCs either by exogenous or endogenous means. The main source of exogenous NOCs includes meats that have been processed, smoked or cured (Bouvard et al., 2015). Alternatively, NOCs can be produced endogenously in the human gut from the intake of nitrite: amino acids are converted to amines *via* bacterial decarboxylation, followed by n-nitrosation in the presence of nitrite as nitrosating agent to generate NOCs (Parnaud et al., 2000; Fahrer and Kaina, 2013).

NOCs are multi-site carcinogens that can form DNA adducts—the outcome of covalent binding between reactive electrophilic species and the nucleophilic sites in DNA (Ewa and Danuta, 2017). Malignant transformation is initiated when N-nitrosamines form diazomethane by the activation of p450 isoenzymes (CYP2E1), resulting in the formation of O^6^-carboxymethyl guanine (O^6^-CMG) and O^6^-methylguanine (O^6^-MeG) DNA adducts, which, when unrepaired, could induce G:C→A:T transition mutation that is typically detected in the K-ras gene associated with human CRC (Fahrer and Kaina, 2013). Red meat was shown to be able to stimulate the production of O^6^-CMG (Lewin et al., 2006; Hemeryck et al., 2016) and O^6^-MeG (Le Leu et al., 2015). The formation of DNA adducts may account for the association between dietary NOC intake and rectal cancer (Loh et al., 2011; Zhu et al., 2014).

##### The Interaction Between N-Nitroso Compounds and Gut Microbiota

Studies have highlighted the involvement of gut microbiota in the production of NOCs (Engemann et al., 2013; Kobayashi, 2018; Yoon and Kim, 2018). Generally, the population of these NOC-producing bacteria is low in healthy individuals, but excessive dietary nitrate and nitrite intake will facilitate the growth of NOC-producing bacteria as nitrate or nitrite are used as electron acceptors during NOC production (Kobayashi, 2018). The resulting microbial dysbiosis can potentially participate in colon carcinogenesis, leading to an elevated CRC risk (Sobhani et al., 2013; Jahani-Sherafat et al., 2018; Zou et al., 2018). The presence of nitrate generated through bowel inflammatory responses was associated with an enrichment of *E. coli* in the large intestine of colitis mice (Winter et al., 2013). Several studies have associated a significantly higher prevalence of pathogenic strains of *E. coli* (usually belong to phylogenetic group B2 or D) with CRC (Buc et al., 2013; Bonnet et al., 2014; Kohoutova et al., 2014; Dejea et al., 2018). Nonetheless, it remains unclear whether the bloom of *E. coli* caused by nitrate or NOCs can contribute to the initiation of intestinal inflammation or colon carcinogenesis.

##### Heterocyclic Amines and Polycyclic Aromatic Hydrocarbons

When muscle meat is cooked at high temperature, its creatinine or creatine, amino acids, and sugars are converted into heterocyclic amines (HCAs) (Helmus et al., 2013). The common types of HCA in meats cooked at high temperature are 2-amino-1-methyl-6-phenylimidazo(4,5-*b*) pyridine (PhIP), 2-amino-3,8-dimethylimidazo(4,5-*f*) quinoxaline (MeIQx), 2-amino-3,4-dimethylimidazo(4,5-f)quinoline (MeIQ), 2-amino-3,4,8-dimethylimidazo(4,5-*f*) quinoxaline (DiMeIQx), and amino-3-methylimidazo(4,5-f) quinolone (IQ). PhIP, MeIQ, and MeIQx have been categorized by International Agency for Research on Cancer (IARC) as possible human carcinogens (group 2B) while IQ has been classified as probably carcinogenic to humans (group 2A) (Cascella et al., 2018). A colonoscopy-based study among Japanese reported a high MeIQ but not MeIQx and PhIP exposure to be positively associated with an increased risk of CRC in females (Budhathoki et al., 2015). However, PhIP intake but not MeIQx and DiMeIQx showed a significantly associated risk of colorectal adenoma (CRA) in an European population-based study (Rohrmann et al., 2009). Conversely, other studies have reported that a higher intake of any type of HCA (PhIP, MeIQx, and DiMeIQx) had significant associated risk for CRA (Barbir et al., 2012; Chiavarini et al., 2017). A study also reported a significant association between CRC risk and red-meat derived, but not white-meat derived HCAs and PAHs (Helmus et al., 2013). Although the definite contribution of each HCA type to colonic neoplasm remains tentative, the findings so far converge to imply that HCAs as a whole are associated with an increased CRC risk, as corroborated by mice studies (Cheung et al., 2011; Lee et al., 2013).

Incomplete combustion of organic materials from industrial processing and domestic cooking at high temperature produces polycyclic aromatic hydrocarbons (PAHs), which could contaminate food when they are in contact. Thus, meat cooked over an open flame is highly prone to PAH contamination (Zelinkova and Wenzl, 2015). Similar to HCA, PAH, primarily benzo(*a*)pyrene (B(*a*)P), could also increase the risk of CRA in humans (Sinha et al., 2005; Chiavarini et al., 2017). It has been shown experimentally that B(*a*)P could bind directly to DNA to form DNA adducts, as well as induce oxidative and nitrosative stress along with increased expression of pro-inflammatory cytokines and dysregulated wnt/β-catenin signaling in mice colon (Diggs et al., 2013; Ajayi et al., 2016). Moreover, there was a synergistic genotoxic effect of HCA and PAH with a five-fold increase in PhIP-derived DNA adducts when B(*a*)P and PhIP were used in combination compared to HCA alone (Jamin et al., 2013).

##### The Interaction Between Heterocyclic Amine and Polycyclic Aromatic Hydrocarbon and Gut Microbiota

Exposure to PhIP (Defois et al., 2018) and B(*a*)P (Defois et al., 2017) could shift the volatile pattern of human fecal microbiota, indicating a deviation in the microbial metabolic activities that may in the long run disrupt gut homeostasis. Of note, these studies exposed microbiota to doses of HCA and PAH that were higher than expected daily consumption for a very short time (24 h), making the results less reflective of the actual effect of chronic HCA and PAH exposure. In murine models, B(*a*)P also induced a shift in the abundance and composition of the gut microbiota, in addition to colonic inflammation (Khalil et al., 2010; Ribière et al., 2016). On the other hand, human colon microbiota could directly induce the bioactivations and transformations of PAH into estrogenic metabolites, influencing the toxicity of PAH (Van de Wiele et al., 2005). Human gut microbiota with beta-glucuronidase and glycerol/diol dehydratase activity could transform HCA to HCA-M1, mitigating HCA-associated carcinogenic risk (Zhang et al., 2019). Specifically, *Eulonchus halli* were able to convert PhIP to PhIP-M1, whose mutagenic potency is only 5% of that of PhIP (Fekry et al., 2016). Just as HCA and PAH can induce dysbiosis to exert detrimental effect in the human body (Khalil et al., 2010; Ribière et al., 2016; Defois et al., 2017; Defois et al., 2018), there are some gut microbiota that could reduce the detrimental effects of dietary HCA and PAH (Van de Wiele et al., 2005; Fekry et al., 2016; Zhang et al., 2019).

##### Trimethylamine-N-Oxide

Trimethylamine-N-oxide (TMAO) is a gut microbiota-dependent metabolite of saltwater fish, eggs, dairy products, and especially, red meat (Velasquez et al., 2016; Chan et al., 2019; Wang et al., 2019). In the human gut, TMAO precursors including dietary choline, phosphatidylcholine, betaine, and L-carnitine undergo gut microbial degradation to be converted into trimethylamine (TMA). TMA is absorbed and delivered to the liver *via* the portal circulation, and subsequently reacts with hepatic flavin monooxygenase, primarily FMO3, to produce TMAO (Velasquez et al., 2016; Subramaniam and Fletcher, 2018; Zou et al., 2018).

Elevated TMAO level is associated with a higher mortality risk of chronic kidney disease (Tang et al., 2015; Missailidis et al., 2016), major adverse cardiovascular events such as coronary artery disease (Senthong et al., 2016), myocardial infarction (Tang et al., 2013; Suzuki et al., 2020), heart failure (Tang et al., 2014; Troseid et al., 2015), and liver cancer (Liu et al., 2018b). Besides, an epigenetic interaction network analysis indicated the role of TMAO on colon carcinogenesis, as many genetic pathways implicated in colon carcinogenesis were shared by TMAO (Xu et al., 2015). From the Women’s Health Initiative (WHI) Observational Study, a higher plasma choline concentration was found to be associated with a greater risk of rectal cancer; women with low plasma vitamin B12 in particular, showed significant association between plasma TMAO level and CRC risk (Bae et al., 2014). A male matched case-control study has indicated that a higher serum concentration of choline was significantly associated with a three-fold increase CRC risk (Guertin et al., 2017). However, other studies have reported a null (de Vogel et al., 2011) and even an inverse (Nitter et al., 2014) association between plasma choline concentration and CRC risk.

TMAO has been found to promote inflammatory gene expression in both human aortic endothelial cells and smooth muscle cells *via* the activation of nuclear factor‐κB (NF‐κB) signaling (Seldin et al., 2016); its concentration was also positively identified to be associated with the concentration of tumor necrosis factor alpha (TNF-α) and its soluble receptors, both of which constitute a key regulator of inflammatory responses (Rohrmann et al., 2015). Additionally, TMAO could upregulate the expression of proinflammatory molecules such as IL-6, CXCL1, and CXCL2 in *H. pylori* infected-gastric epithelial cells, demonstrating the possible synergistic effect of TMAO and *H. pylori* in the development of gastritis or gastric ulcers (Wu et al., 2017). TMAO could also activate NLRP3 inflammasome and the production of reactive oxygen species (ROS) in fetal human colon cells by inhibiting ATG16L1-induced autophagy, a process that is vital for the regulation of inflammation (Yue et al., 2017). The roles of TMAO in inflammatory process implicated by these findings provide a plausible mechanism by which TMAO could contribute to colon carcinogenesis.

##### The Interaction Between Trimethylamine-N-Oxide and Gut Microbiota

The composition of intestinal microbiota influences the production of TMAO from its precursors (Chan et al., 2019). Following antibiotics treatment that suppressed intestinal microbiota, dietary supplementation of TMAO precursors failed to enhance TMAO synthesis in mice (Koeth et al., 2013) or healthy adults (Tang et al., 2013), implying the dependence of TMAO production on the metabolism of gut microbiota. Besides, serum TMAO level could be lowered by a natural compound known as berberine *via* altering the composition of gut microbiota, in particular Firmicutes and Verrucomicrobia, leading to an anti-atherosclerotic effect (Shi et al., 2018). When compared to a non-TMA producing bacterial species, TMA-producing species brought about a significantly higher serum TMAO in germ-free mice (Romano et al., 2015). In a study involving healthy young men, those with a higher Firmicutes to Bacteroidetes ratio demonstrated a greater response to dietary TMAO, implying the influence of gut microbiota composition on TMAO production (Cho et al., 2017). There are evidences implying the role of gut microbiota as a mediator of the association between diet, TMAO, and diseases, with alteration in the composition or structure of intestinal microbiota influencing TMAO production and hence, affecting the risk of colonic lesions (Koeth et al., 2013; Tang et al., 2013; Romano et al., 2015; Cho et al., 2017; Shi et al., 2018; Chan et al., 2019). Of note, although most of the intestinal microbiota promote TMAO production from its precursors*, Eubacterium limosum* was shown to be able to metabolize TMA precursors through carnitine demethylation to form a product that cannot be readily converted into TMA, revealing the potential of this gut bacterium in reducing TMAO level in the gut (Kountz et al., 2020).

##### Heme Iron

Red meat but not white meat, is associated with an elevated risk of CRC, and this has been linked to heme iron (Cross et al., 2010; Bastide et al., 2011; Oostindjer et al., 2014). Heme is enriched in hemoproteins including myoglobin, hemoglobin and cytochrome. Red meat in relation to white meat, has higher concentration of myoglobin, with a 10-fold higher heme content (Bastide et al., 2011). Heme-induced CRC risk is linked to two reactions catalyzed by heme, namely N-nitrosation and lipoperoxidation. The former is characterized by the decarboxylation of amino acids by nitrosating agents to form NOCs (Bastide et al., 2011). In comparison with diet containing a negligible amount of heme, heme-rich red meat and processed meat could significantly increase fecal NOCs level, confirming the contribution of heme to endogenous NOC production (Joosen et al., 2009). Processed meat generally contains a higher amount of NOCs than fresh meat as heme iron in processed meat products is nitrosylated (curing salt contains nitrite or nitrate), resulting in the formation of nitrosyl heme (Cammack et al., 1999). In a cohort study, both nitrosylated and non-nitrosylated heme iron were found to be associated with CRA risk, but a higher risk was associated with the former, implying its higher carcinogenicity. This provides a possible explanation for the stronger link between CRC risk and processed meat intake than fresh meat intake (Bastide et al., 2016). Additionally, consumption of a large amount of red meat also results in the formation of N-nitrosothiols, which together with nitrosyl heme, leads to the accumulation of carboxymethylated adducts (Steinberg, 2019). Indeed, the mutagenicity of NOCs has been attributed to the formation of DNA adducts such as O^6^-CMG and O^6^-MeG, which could potentially accelerate the malignant transformation in the colon (Lewin et al., 2006; Bugni et al., 2009; Le Leu et al., 2015).

Lipoperoxidation is characterized by the free-radicals attack of membrane lipids which gives rise to aldehydes such as malondialdehyde (MDA) and 4-hydroxynonenal (4-HNE) (Bastide et al., 2011). Heme-induced lipoperoxidation can result in an increased fecal thiobarbituric acid reactive substances (TBARs). Previously, an increased fecal TBARs level was observed in mice (Martin et al., 2015) and human subjects (Pierre et al., 2013) who were given heme-rich diets, thus, substantiating the role of heme in promoting lipoperoxidation. In a recent study, *ex vivo* trapping of aldehyde counteracted inflammation and DNA damage in murine colonic epithelial cells, attributing these heme-induced deleterious effects to lipoperoxidation (Martin et al., 2019b).

In addition to NOCs and aldehydes, hemin, a porphyrin with iron bound to chloride derived from a heme group, is also related to colon tumor growth (Kim et al., 2019a). Hemin could exert cytotoxic effect on colonic epithelial cells *via* the production of ROS such as hydrogen peroxide (Ishikawa et al., 2010; Gemelli et al., 2014). Hemin could also mimic the effect of freeze-dried ham in inducing aberrant crypt foci and mucin depleted foci in rodent models, both of which are the precancerous lesions of the colon (Pierre et al., 2010).

##### The Interaction Between Heme and Gut Microbiota

The consumption of heme-rich diet was shown to alter the composition of gut microbiota in mice, which was exemplified by a reduction of Firmicutes and Deferribacteres, in addition to an increase of Proteobacteria or Bacteroidetes (Ĳssennagger et al., 2012; Constante et al., 2017; Martin et al., 2019b). The dysbiosis could reduce the colonic level of butyrate, a short chain fatty acid important for the digestive health, leading to adenoma formation (Constante et al., 2017). Interestingly, the dysbiosis could be ameliorated by the supplementation of calcium to heme-rich diet (Martin et al., 2019b). Other study has demonstrated the ability of gut microbiota to modulate the lipoperoxidation associated with heme-induced colon carcinogenesis (Martin et al., 2015). An antibiotic treatment was able to prevent epithelium damages and hyperproliferation in mice fed with heme-rich diet compared to antibiotics absence, substantiating the involvement of gut microbiota in enhancing heme-induced hyperproliferation and deteriorating the colonic mucus barrier (Ijssennagger et al., 2015).

#### Other Protein Sources

Fish consumption has no association (Engeset et al., 2007; Lee et al., 2009; Sugawara et al., 2009; Pham et al., 2013) or an inverse association (Wu et al., 2012; Aglago et al., 2019) with CRC risk. The association between egg intake and CRC risk is inconsistent, with some studies reporting a positive association (Aune et al., 2009; Lee et al., 2009) while others reported a null association (Franceschi et al., 1997; Järvinen et al., 2001). On the other hand, dairy products are associated with a decreased colon cancer risk (Huncharek et al., 2008; Aune et al., 2012), particularly cheese and milk (Lee et al., 2009; Aune et al., 2012; Murphy et al., 2013; Barnung et al., 2019).

Several studies have indicated the CRC-promoting effects in red and processed meat but not in other protein sources which also contain the CRC-inducing components found in red and processed meats. The other protein sources such as fish, eggs, and dairy products also contain the precursors of TMAO, choline, phosphatidylcholine, and L-carnitine (Velasquez et al., 2016; Subramaniam and Fletcher, 2018), while baked or grilled fish contain HCA and PAH (Alisson-Silva et al., 2016). The lack of a link between CRC and the consumption of protein sources other than meat may be explained by various health-promoting dietary components. For instance, studies have attributed the protective effect of fish due to its omega-3 polyunsaturated fatty acids (n-3 PUFAs) (Pietrzyk, 2017; Aglago et al., 2019). n-3 PUFAs are known to exhibit an anti-inflammatory effect through the mediation of the expression of inflammatory genes, or by modulating intracellular signaling pathways that regulate T-cell activation, leading to a decreased risk of colon carcinogenesis (Chapkin et al., 2007; Wall et al., 2010). Calcium from dairy products is found to inhibit tumorigenesis in human colon cancer cells and CRC mouse models (Wang et al., 2011; Ju et al., 2012). The CRC-protective effect of calcium has been linked to its ability to reduce lipid peroxidation induced by heme (Hemeryck et al., 2016; Martin et al., 2019b), and to restore the upregulation of inflammatory genes induced by Western diet in the human colons (Protiva et al., 2016). Importantly, only calcium from dairy products, but not the non-dairy products, was associated with a decreased CRC risk (Murphy et al., 2013).

Although it is unclear whether certain health-promoting dietary components could counteract the harmful effects of CRC-associated dietary components, it is evident that colon carcinogenesis is not attributed to a single nutrient but a combination of them, such that the dietary impact of a particular food chemical may be influenced by the absence or presence of another (Pietrzyk, 2017). This may explain why protein sources containing the same carcinogens exhibit opposite effects in CRC.

### Carbohydrates

Carbohydrates are macronutrients categorized as simple or complex carbohydrates based on their degree of polymerization (Ludwig et al., 2018). Simple carbohydrates such as monosaccharides, disaccharides, and oligosaccharides are found in sugar-sweetened food and beverage or refined plant-based food, whereas complex carbohydrates including polysaccharides such as dietary fibers and resistant starches are enriched in unrefined plant-based food (Hansen and Sams, 2018; Ludwig et al., 2018). Carbohydrate polymer length influences not only its digestion and absorption into the human body, but also the subsequent impacts on body functions (Ludwig et al., 2018). Thus, simple and complex carbohydrates will be discussed separately in terms of their contributions to CRC.

#### Complex Carbohydrates

The contribution of dietary fibers in reducing CRC risk has been well established in case-control studies (Dahm et al., 2010b; Luo et al., 2015), meta-analysis (Ben et al., 2014; Gianfredi et al., 2018) and European Prospective Investigation into Cancer and Nutrition (EPIC) studies (Murphy et al., 2012; Bradbury et al., 2014). The protective effect of dietary fibers is attributed to short chain fatty acids (SCFA) such as acetate, propionate, and butyrate, which are formed following the fermentation of fibers by gut microbiota along the colon tract (Wu et al., 2018). Among the different types of SCFA, butyrate has been most extensively studied for its protective efficacy against CRC. Its inhibitory effect against human colon cancer cell proliferation was shown superior compared with acetate and propionate (Zeng et al., 2020).

Butyrate exerts its anti-proliferative effect in CRC cells *via* several mechanisms. Firstly, butyrate decreases the expression of neuropilin-1, a receptor of vascular endothelial growth factor (VEGF) that is commonly upregulated in colon cancer cells. As VEGF is a key regulator of angiogenesis, the downregulation of neuropillin-1 exerts an inhibitory effect on the expansion of colon cancer cells (Yu et al., 2010; Yu et al., 2011). Second, butyrate induces apoptosis and suppresses the proliferation and invasion of CRC cells by regulating the expression of microRNA such as miR-92a (Hu et al., 2015) and miR-203  (Han et al., 2016). Thirdly, butyrate reduces motility of CRC cells by inhibiting the Akt/ERK signaling pathway (Li et al., 2017), revealing the potential of butyrate as part of the therapeutic strategy for blocking metastatic CRC. The protective effect of butyrate was substantiated in another study that reported a significant downregulation of free fatty acid receptor 2 (FFAR2), a butyrate receptor, in human colon cancer tissues than in healthy tissues, while mice deficient in FFAR2 also developed significantly more colon polyps than wild-type mice (Sivaprakasam et al., 2016).

Interestingly, butyrate stimulates cell growth in normal colonocytes but exerts an anti-proliferative effect in cancerous colonocytes. Such discrepancy in function was termed the “butyrate paradox.” The “Butyrate paradox” is explained by the “Warburg effect” which describes the differential metabolism of butyrate in cancerous and non-cancerous cells: non-cancerous cells mainly undergo oxidative metabolism, using butyrate as a primary energy source, whereas cancerous cells favor glycolysis as a means of metabolism, resulting in an inefficient metabolism of butyrate (Donohoe et al., 2012). In cancerous colonocytes, butyrate also downregulates its own oxidation by reducing the expression of short chain acyl-CoA dehydrogenase (SCAD), an enzyme that is responsible for butyrate oxidation (Han et al., 2018). Accordingly, butyrate could accumulate and function as a histone deacetylase inhibitor to stall the proliferation of cancer cells (Donohoe et al., 2012). A corroborative study has shown that butyrate suppressed the proliferation of the underlying colonic stem cells by inhibiting histone deacetylase only in damaged colonocytes but not in normal colonocytes (Kaiko et al., 2016). Hence, although butyrate serves as a primary source of energy for the proliferation of normal colon cells, it inhibits the growth of cancerous colonocytes, which highlights the tremendous advantages of dietary fiber intake.

##### The Interaction Between Complex Carbohydrates and Gut Microbiota

Studies have provided evidence that dietary carbohydrates are able to modify gut microbiota and hence, could modulate the physiological conditions in the colonic environment. For instance, children from rural African villages whose diet consists of mostly fibers showed a significant enrichment in Bacteroidetes compared with Italian children. Within the Bacteroidetes phylum, several cellulose-degrading bacteria from the genus *Prevotella* were found to be completely lacking in European children (De Filippo et al., 2010). A dominance of *Prevotella*, along with depletion of *Bacteroides* was also found in the gut microbiota of Bangladeshi children compared with children from the United States (Lin et al., 2013), as well as in native Africans compared with African Americans (Ou et al., 2013). Indeed, diet serves as a driving force for shaping the intestinal microbiome, where long-term adherence to fiber-rich food leads to the abundance of *Prevotella*, whereas adherence to Western diet low in fiber results in abundance of *Bacteroides* (Wu et al., 2011; De Filippis et al., 2016; Gorvitovskaia et al., 2016). *Bacteroides* species, in particular *Bacteroides fragilis*, were found to be prevalent in the colonic mucosa of CRC patients (Boleij et al., 2015; Haghi et al., 2019). Their ability to induce DNA damage *via* ROS production in colonic epithelial cells (Goodwin et al., 2011) and colitis along with colon tumors in mice (Wu et al., 2009) has also been documented, indicating the possible role of *Bacteroides* species in promoting colonic neoplasm in individuals with low fiber intake.

On the other hand, although *Prevotella* is frequently linked to high fiber diet, strains such as *Prevotella copri* and *Prevotella intestinalis* have been linked to HIV-associated (Dillon et al., 2016) or intestinal inflammatory conditions (Scher et al., 2013; Iljazovic et al., 2020). Given Prevotella constitutes a large genus with great species diversity, more studies are warranted to clarify its beneficial or detrimental roles in the human body, including the mechanisms underlying its interaction with dietary components to clarify its role in the pathogenesis of CRC (Ley, 2016; Larsen, 2017). Other studies have reported the association of dietary fiber with a lower risk for *F. nucleatum*-positive CRC but not for *F. nucleatum*-negative CRC, suggesting the role of *F. nucleatum* as a mediator of the association between dietary fibers and colorectal neoplasms (Mehta et al., 2017; Liu et al., 2018a). Whether or not this hypothesis can be generalized to *Bacteroides* or *Prevotella* remains to be explored.

As discussed above, the protective effect of dietary fibers could be largely attributed to butyrate production. Of note, the fermentation of dietary fibers to produce butyrate is largely mediated by gut microbiota including *Faecalibacterium prausnitzii*, *Eubacterium rectale*, *Roseburia faecis*, *E. halli*, and others (Baxter et al., 2019). Thus, it is relevant to propose that the gut microbiome could influence the dietary impact of butyrate. A smaller population of butyrate-producing bacteria such as *Roseburia*, *Clostridium*, and *Eubacterium* was observed in advanced CRA patients along with a lower production of butyrate, highlighting the importance of gut microbiota in modulating the production of butyrate to protect hosts from colonic neoplasm (Chen et al., 2013). Conversely, butyrate could change the composition of gut microbiota to attenuate the detrimental effect of high-fat diet, resulting in an improve intestinal barrier and attenuated obesity or steatohepatitis (Zhou et al., 2017; Fang et al., 2019). Similarly, sodium butyrate supplementation was shown to beneficially restore the dysbiosis in CRC liver metastasis mouse model, resulting in a better host immune system characterized by an increase in natural killer T cells and T helper 17 cells (Ma et al., 2020). Hence, the interaction between butyrate and gut microbiota appears to be bidirectional. More investigations would need to be conducted to define the mechanistic link between dietary butyrate, gut microbiota, and CRC.

#### Simple Carbohydrate

A conclusive link between high sugar intake and CRC risk in healthy humans has not been reported by cohort studies (Galeone et al., 2012; Tasevska et al., 2012; Pacheco et al., 2019), but a moderately heightened risk did emerge in smaller case-control studies (Pou et al., 2012; Wang et al., 2014; Chun et al., 2015). Nonetheless, the detrimental effect of simple carbohydrates on CRC is more evident in animal studies. Sucrose supplementation aggravated colonic inflammation in colitic rats by inducing DNA changes in the colon mucosal cells (Mahmoud, 2011). Ingestion of high-fructose corn syrup could enhance colon tumorigenesis *via* the action of ketohexokinase, a fructose-converting enzyme that changes the tumor cell metabolism to increase the production of fatty acids needed for tumor growth. Importantly, the enhanced tumor growth was observed in the absence of obesity, supporting the detrimental role of simple sugars in the etiology of CRC independently from their effect on obesity (Goncalves et al., 2019). This finding points to the consequences of consumption of simple sugars, especially of high-fructose corn syrup that is widely used as a sweetener in carbonated drinks, condiments, and baked foods (Payne et al., 2012).

Simple carbohydrates, especially monosaccharides, are easily hydrolyzed and absorbed in the small intestine, resulting in a rapid rise in blood glucose level. Although a consistent link between total sugar intake and CRC risk has not been established, a number of studies did find a positive association between glycemic index (GI) and glycemic load (GL) with CRC risk (Gnagnarella et al., 2008; Sieri et al., 2015). The GI measures the elevation in blood sugar level following consumption of a particular food independent of quantity, while GL takes into account of both GI and the quantity of available carbohydrates in a portion of food consumed (Vega-López et al., 2018). Interestingly, GI has been significantly linked to CRC risk more frequently than GL (George et al., 2009; Choi et al., 2012; Turati et al., 2015). Two studies reported that high carbohydrate intake from high GI food was significantly associated with increased CRC risk, but high carbohydrate intake from low GI food was significantly associated with decreased CRC risk, indicating the greater dependence of CRC risk on the ability of carbohydrate-rich food to post-prandially raise blood glucose level rather than the overall amount of carbohydrates consumed (Sieri et al., 2015; Sieri et al., 2017). In fact, hyperglycemia (high blood sugar level) and hyperinsulinemia (high insulin level) resulting from high GI-diet could contribute to the initiation or progression of CRC through the induction of DNA damage in colon cells (Othman et al., 2013; Othman et al., 2014), or the action of insulin-like growth factor (Aleksandrova et al., 2013) which has a known role in the pathogenesis and progression of CRC (Vigneri et al., 2015; Kasprzak and Adamek, 2019). Other etiological hypotheses for the CRC-promoting effect of high GI/GL diet include insulin resistance, oxidative stress and abnormal sex hormone production (Sieri et al., 2017; Li et al., 2019), but these mechanisms have not been extensively studied.

##### The Interaction Between Simple Carbohydrates and Gut Microbiota

The association between simple carbohydrate consumption and microbiota-mediated CRC risk remains poorly documented. Martinez-Medina et al. (2014) reported an increased colonization of adherent-invasive *E. coli* and mucin-degrading bacterium *Ruminococcus torques* in the gut of mice fed with high-fat, high-sugar Western diet. The change in microbiota composition subjected the mice to an increased susceptibility to inflammation, as reflected by the decreased mucus thickness and an increased release of pro-inflammatory cytokines (Martinez-Medina et al., 2014). Of note, the study examined the combined effect of a high-fat and a high-sugar diet, hence it cannot be concluded whether the microbial dysbiosis was induced by fat or sugar exposure. Nevertheless, it has been postulated that a prolonged exposure to fructose and sugar substitutes through a Western diet can subject gut microbiota to extensive conditioning, leading to the formation of “Western gut microbiome” characterized by low microbial genetic and phylogenic diversity (Payne et al., 2012; Segata, 2015). Supporting this hypothesis, studies have shown that individuals living in non-urban settings harbor a gut microbiome of greater diversity and complexity, relative to individuals from urban-industrialized settings (Obregon-Tito et al., 2015; Rampelli et al., 2015). The loss or depletion of certain gut microbiota may in the long run lead to microbial dysbiosis or aberrant host-microbe interactions, and ultimately, disorders (Payne et al., 2012).

### Fat

Epidemiological studies examining the link between dietary fat intake and CRC risk are sparse and to date, have not drawn a definitive conclusion regarding the CRC-promoting effect of dietary fats. While total dietary fat intake is infrequently linked to CRC risk (Dahm et al., 2010a; Williams et al., 2010; Kim and Park, 2018), an elevated CRC risk has been detected among individuals with a high saturated fat intake (Chun et al., 2015; Tayyem et al., 2015; Kim et al., 2017b). In contrast, consumption of n-3 polyunsaturated fat (n-3 PUFA) has been inversely associated with CRC (Zhong et al., 2013; Kim and Kim, 2020). A more consistent link between fat intake and colon carcinogenesis has been inferred from mice studies, that showed an associated increased risk due to an altered expression of inflammatory mediators (Padidar et al., 2012; Day et al., 2013).

Investigations of the dietary fat-associated CRC risk often cite bile acids as being responsible for increasing CRC risk. Human primary bile acids, namely cholic acid (CA) and chenodeoxycholic acid (CDCA), are synthesized in the liver from cholesterol. Conjugated with glycine and taurine, the primary bile acids are secreted into the hepatobiliary system and enter the duodenum to emulsify dietary fats after a meal. Subsequently, the majority of the primary bile acids will be deconjugated and reabsorbed, but a small amount can escape and enter the colon, where they are biotransformed by colonic bacteria *via* 7α-dehydroxylation to form secondary bile acids such as deoxycholic acid (DCA) and lithocholic acid (LCA). Most human gut microbiota that participate in the 7α-dehydroxylation belong to the genus *Clostridium* (Ocvirk and O’Keefe, 2017; Zou et al., 2018).

Given bile acids are cholesterol derivatives, their synthesis is likely to be promoted by a high-fat diet that is also rich in cholesterol. A high-fat diet will upregulate bile discharge for the emulsification of excessive dietary fats, which further raises the bile acid level (Ajouz et al., 2014). A study reported an increased fecal bile acid concentration in mice fed with high-fat, low-fiber Western diet, which was observed along with a defective bile acid transport and an increased colon tumor number (Dermadi et al., 2017). Accumulation of fecal bile acid was also observed in DCA-treated mice, along with a significantly increased intestinal inflammation (Xu et al., 2020). Extending to human studies, African Americans who consumed a high-fat diet were shown to have a 3–4 times higher level of secondary bile acids than native Africans who subsisted on a low-fat diet, providing a possible explanation for the much higher CRC risk among African Americans (Ou et al., 2012). In the follow-up study that involved switching African Americans to a high-fiber, low-fat diet for 2 weeks, the dietary change led to a suppressed secondary bile acid synthesis and showed a remarkable decrease in colonic mucosal inflammation and proliferation biomarkers of cancer risk (O’Keefe et al., 2015).

Although the exact mechanism of how bile acids induce the pre-cancerous state of colonocytes is unclear, it has been hypothesized that bile acids as cholesterol derivatives with detergent-like properties, could damage the intestinal epithelium when they are present at high concentration. Such destruction will trigger inflammation and a compensatory hyperproliferation of undifferentiated cells, which drives their transition into a precancerous state (Nguyen et al., 2018). Moreover, DCA and LCA could induce cancer stem cell growth in the colonic epithelium *via* the modulation of M3R and Wnt/β-catenin signaling. Given cancer stem cells are notorious for their ability to initiate and sustain tumor growth or proliferation, this finding provides a probable mechanism on how bile acids promote CRC (Farhana et al., 2016). Interestingly, it has been hypothesized that bile acid could exert a dichotomous effect on the apoptosis of colonocytes (Nguyen et al., 2018): a short-term exposure to high concentration of bile acid was able to induce apoptosis, primarily *via* the production of ROS (Ignacio Barrasa et al., 2011), whereas a prolonged exposure gave rise to colon carcinogenesis (Bernstein et al., 2011), which can be attributed to the inhibited accumulation of tumor suppressor p53 (Qiao et al., 2001), or the activation of PI3K/Akt signaling (Raufman et al., 2008). The effect of bile acids on CRC development was shown to be mediated by the farnesoid X receptor (FXR), a primary bile acid nuclear receptor. One study reported that inactivation of FXR could increase cancer risk by inducing bile acid dysregulation (Dermadi et al., 2017). Conversely, in another study, the ability of DCA to compromise colonic epithelial restitution appeared to be mediated by the activation of FXR, and such effect was linked to colonic barrier dysfunction and intestinal inflammation, both of which could increase susceptibility to CRC (Mroz et al., 2018). Although the role of FXR remains incompletely defined, these studies pinpoint the importance of FXR in mediating bile acid-associated CRC risk.

Although DCA and LCA are notorious for their tumorigenic effect especially in the course of CRC development, it is vital to note that not all bile acids could cause adverse effects. Ursodeoxycholic acid (UDCA), a secondary bile acid used as the first-line therapy for primary biliary cirrhosis, is found to have profound chemopreventive effect against colorectal carcinogenesis (Serfaty et al., 2010). The antiproliferative action of UDCA in colon cancer cells has been well documented and attributed to its ability to regulate the production of ROS (Kim et al., 2017a), to suppress the pro-proliferative c-Myc proteins (Peiro-Jordan et al., 2012), or to sustain the hyperphosphorylation of ERK1 kinase (Krishna-Subramanian et al., 2012). UDCA could also reverse DCA-induced effect on colonic epithelial cells by inhibiting DCA-induced secretion of epithelial defensins (Lajczak et al., 2017). Evidently, while other secondary bile acids drive CRC progression, UDCA emerges as a potential chemopreventive agent for CRC. Their differential actions on CRC-related inflammatory signals or growth factors are likely to account for such contrasting effect (Serfaty et al., 2010).

#### The Interaction Between Dietary Fat and Gut Microbiota

Several studies have associated the prolonged intake of high-fat diet with a shift in the relative proportion of Firmicutes to Bacteroidetes (F/B ratio), where Firmicutes dominate at the expense of Bacteroidetes in most cases (Murphy et al., 2010; Islam et al., 2011; Taira et al., 2015). Given the shift in the F/B ratio has been repeatedly linked to obese individuals (Ferrer et al., 2013; Kasai et al., 2015; Koliada et al., 2017), it could be contended that the dysbiosis is more likely to be associated with the host obese state rather than the high-fat diet itself. However, it was found that even in the absence of an obese genotype, mice fed with high-fat diet demonstrated profound changes in microbial communities characterized by increase in Firmicutes and decrease in Bacteroidetes (Hildebrandt et al., 2009). A high-fat diet could also promote tumorigenesis in the murine small intestine independently of obesity by inducing a dysbiosis that was associated with an attenuated antimicrobial host defense (Schulz et al., 2014). These findings collectively show that microbial dysbiosis is inducible by high-fat diet alone, and such dysbiosis could promote colon carcinogenesis independently of obesity.

Moreover, a high-fat diet could increase the proportion of the phylum Actinobacteria with an accompanying increase in the expression of proinflammatory cytokines and a decrease in tight junction proteins (Kim et al., 2019b). Given Actinobacteria are known mucin-degrading bacteria (Tailford et al., 2015), an increase in abundance could induce gut barrier impairment and subsequent colonic inflammation, which may help explain the CRC-promoting effect of dietary fat in the colon. In mice, a high-fat diet also diminished the beneficial gut microbiota including *Bifidobacterium*, *Lactobacillus*, and *Akkermansia* (Kim et al., 2012; Lecomte et al., 2015; He et al., 2018), all of which play significant roles in mediating the intestinal metabolism and immunity (Naito et al., 2018). The high-fat diet-induced reduction of these beneficial gut microbiota could possibly increase the susceptibility to inflammation, facilitating subsequent neoplastic progression.

As conversion of bile acids into secondary bile acids involves the action of gut microbiota, changes in the composition of gut microbiota can potentially influence the metabolic effect of bile acids in the host. A dysbiosis characterized by a decrease in the ratio between *Faecalibacterium prausntizii* and *E. coli* was associated with an impaired bile acid metabolism in patients with inflammatory bowel diseases (IBD), as reflected by their higher level of fecal bile acids (Duboc et al., 2013). In a recent metagenomics analysis, a lower abundance of genes of bile-metabolizing Firmicutes was detected in the gut microbiota of IBD individuals, again linking microbial dysbiosis with the disease state (Das et al., 2019). Of note, the interaction between bile acids and gut microbiota is bidirectional as bile acids serve as antimicrobial agents that help shape the gut microbiome structure (Ridlon et al., 2016). Administration of cholic acid into rats could expand Firmicutes to approximately 95% of the total gut microbiome in relation to 54% in control rats (Islam et al., 2011). DCA treatment could induce a dysbiosis characterized by an increase in opportunistic pathogens and a decrease in *Lactobacillus*, *Lactococcus*, and *Roseburia*, which was linked to intestinal tumorigenesis (Cao et al., 2017). Indeed, while gut microbiota can affect the metabolism of bile acid, bile acid can shape the composition of gut microbiota.

Recently, *Bilophila wadsworthia*, a sulfur-metabolizing microbe that convert dietary sulfur into genotoxic hydrogen sulfide (H_2_S) have been associated with the development of CRC (David et al., 2014; Ijssennagger et al., 2016; Nguyen et al., 2020). Excessive gut-derived H_2_S could break down the disulfide bonds of the mucus bilayer of the gastrointestinal tract and expose gut epithelium to immunogenic luminal bacteria (Ijssennagger et al., 2016). Moreover, it could cause epithelial DNA damage, promote alterations in immune cell populations associated with inflammation and CRC. David et al. (2014) reported that participants fed with a high-meat, high-fat diet for several days showed an increase in *Bilophila*, which coincided with an increased abundance of microbial DNA and RNA encoding for H_2_S-producing enzymes (David et al., 2014). In a large cohort of patients with precancerous polyps and CRC, Yachida et al. (2019) found that DCA concentration was significantly increased in subjects with multiple polypoid (MP, more than three adenomas) adenomas with low grade dysplasia as compared to healthy controls; and *Bilophila wadsworthia* was the only species that was significantly associated with DCA increase in this MP group (Yachida et al., 2019). Nguyen et al. (2020) reported that long-term adherence to a dietary pattern associated with sulfur-metabolizing bacteria in stool was associated with an increased risk of distal CRC in men but how this bacteria contribute to CRC pathogenesis remains unclear (Nguyen et al., 2020).

**Figure 1:**
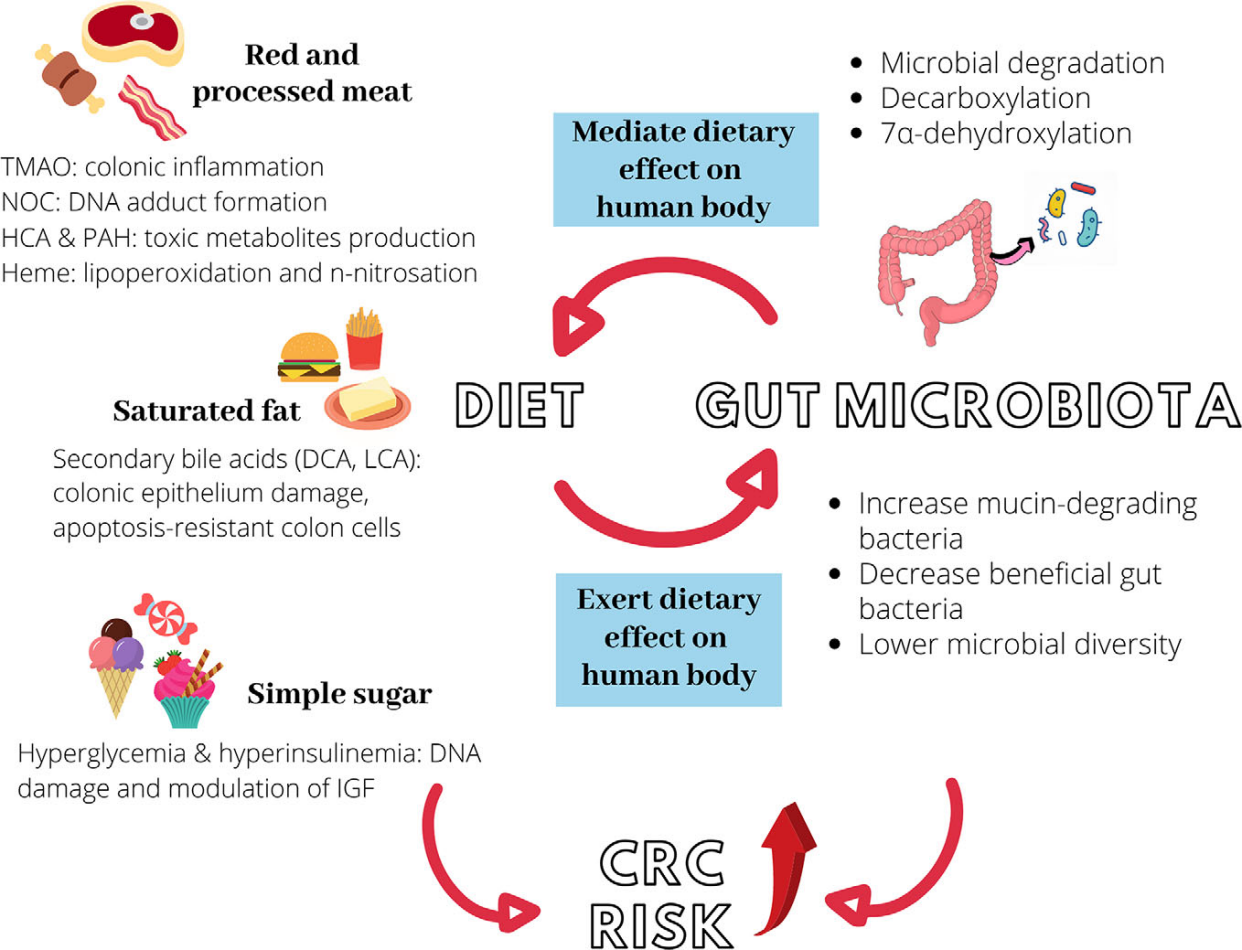
Diet, gut microbiota, and increased colorectal cancer (CRC) risk. Red and processed meat, saturated fat, and simple sugar could independently promote colon carcinogenesis *via* different mechanisms. Similarly, they can induce microbial dysbiosis in the intestine, further elevating CRC risk. Gut microbiota modulates the metabolism of ingested food *via* different processes to produce carcinogenic metabolites such as NOC, TMAO, and secondary bile acids. The interaction between diet and gut microbiota is hence bidirectional. TMAO, trimethylamine-N-oxide; NOC, N-nitroso compounds, HCA, heterocyclic amines; PAH, polycyclic aromatic hydrocarbons; DCA, deoxycholic acid; LCA, lithocholic acid; IGF, insulin-like growth factor.

**Figure 1** illustrates the interactions between diet, gut microbiota, and increased risk of CRC risk. **Table 2** recapitulates the interactions between dietary compounds and gut microbiota that purportedly influence CRC risk.

**Table 2:** Interactions between dietary compounds and gut microbiota that purportedly influence colorectal cancer (CRC) risk.

Food type	Compounds involved	Action of dietary compounds on gut microbiota	References	Action of gut microbiota on dietary compounds	References
Red and processed meat	N-nitroso compounds (NOCs)	Promote the growth of NOC-producing bacteria, leading to disease- causing dysbiosis	(Kobayashi, 2018)	–	–
	Heterocyclic amines (HCAs) and polycyclic aromatic hydrocarbons (PAHs)	Alter the abundance, composition, or metabolic activities of gut microbiota, accompanied by colonic inflammation	(Khalil et al., 2010; Ribière et al., 2016; Defois et al., 2017; Defois et al., 2018)	Bioactivate and transform HCAs and PAHs into compounds with lower toxicity	(Van de Wiele et al., 2005; Fekry et al., 2016; Zhang et al., 2019)
	Trimethylamine-N-oxide (TMAO)	–	–	Involved in TMAO synthesis; microbial composition could define body response toward TMAO intake	(Koeth et al., 2013; Tang et al., 2013; Romano et al., 2015; Cho et al., 2017)
	Heme	Promote the enrichment of Proteobacteria and Bacteroidetes; reduce Firmicutes and Deferribacteres; promote adenoma formation; decrease fecal butyrate level	(Ĳssennagger et al., 2012; Constante et al., 2017; Martin et al., 2019b)	Increase heme-induced lipoperoxidation, hyperproliferation and exacerbation of colonic mucus barrier	(Ijssennagger et al., 2015; Martin et al., 2015)
Dietary fibers	Butyrate	Promote the enrichment of Bacteroidetes, especially Prevotella; restore high-fat diet-induced dysbiosis, resulting in an improved host immune response and intestinal barrier as well as attenuated obesity and steatohepatitis	(De Filippo et al., 2010; Lin et al., 2013; Ou et al., 2013; Zhou et al., 2017; Fang et al., 2019; Ma et al., 2020)	Mediate the fermentation of dietary fiber to form butyrate	(Chen et al., 2013; Baxter et al., 2019)
Simple sugar	–	Promote the enrichment of pathogenic and mucin degrading bacteria; form “Western gut microbiome” characterized by low genetic and phylogenic diversity	(Payne et al., 2012; Martinez-Medina et al., 2014; Segata, 2015)	–	–
Fats	Bile acids	Increase the Firmicutes to Bacteroidetes ratio, which was linked to obesity; promote the enrichment of mucin-degrading Actinobacteria; decrease beneficial gut microbiota such as *Bifidobacterium*, *Lactobacillus*, and *Akkermansia*	(Murphy et al., 2010; Islam et al., 2011; Kim et al., 2012; Lecomte et al., 2015; Taira et al., 2015; Cao et al., 2017; He et al., 2018)	Mediate the conversion of bile acids into secondary bile acids; dysbiosis is associated with impaired bile acid metabolism and inflammatory bowel diseases	(Duboc et al., 2013; Zou et al., 2018; Das et al., 2019)

## The Underlying Molecular Mechanisms of Colon Carcinogenesis Mediated by Gut Microbiota

### Inflammation and Host Defense Mechanism

The connection between inflammation and malignant diseases has been well-established based on evidence from epidemiological, genetic, or pharmacological studies. In the case of colon malignancies, IBDs especially ulcerative colitis and Crohn’s disease exhibit consistent association with an elevated risk of CRC (Choi et al., 2019; Zhou et al., 2019; Olén et al., 2020). IBD-associated CRC is characterized by early onset (Keller et al., 2019), increased formation of synchronous, and poorly differentiated tumors (Reynolds et al., 2017) as well as elevated mortality rate (Sebastian et al., 2014), in relation to sporadic CRC.

Importantly, recent studies have indicated the role of gut microbiota in mediating inflammatory responses during the course of colon carcinogenesis, most of which are through the activation of nuclear factor-κB (NF-κB) signaling pathway. NF-κB are a family of transcription factors that actively regulate genes of various immune and inflammatory responses, and strong evidence has indicated its involvement in the pathogenesis of IBD and IBD-associated cancers (Liu et al., 2017). In a mice study, colon tumors with a higher abundance of *Fusobacterium nucleatum* had a higher nuclear translocation of the p65 NF-κB subunit, representing an increase in the activation of NF-κB pathway (Kostic et al., 2013). Importantly, the tumorigenic property of *F. nucleatum* was indicated as its administration into Apc^Min/+^ mice and human CRC cell lines accelerated the colonic tumorigenesis, which was attributed to the activation of NF-κB that in turn induced miR21 expression (Yang et al., 2017). Analysis of colonic epithelial tissue of CRC patients showed a higher expression of NF-κB in the *S. gallolyticus*-seropositive group compared to the *S. gallolyticus*-seronegative group (Abdulamir et al., 2009). In the follow-up study, the colorectal tissue of *S. gallolyticus*-seropositive CRC patients had a higher expression of IL-1 and COX-2, both of which constitute the products of NF-κB activity (Abdulamir et al., 2010). Likewise, infection of human macrophages with CRC-associated *E. coli* strains promoted a sustained COX-2 expression (Raisch et al., 2015). These studies together underscore the role of gut microbiota together with the involvement of NF-κB during the course of colon carcinogenesis.

The infection of colonocytes by certain gut microbiota can activate a wealth of immune cells that together mediate the colonic inflammatory responses. Enterotoxigenic *B. fragilis* for instance, could induce distal colon tumorigenesis by recruiting polymorphonuclear immature myeloid cells following NF-κB activation (Chung et al., 2018). *Peptostreptococcus anaerobius* interacts with colonic cells *via* α2/β1 integrin, a receptor that is commonly overexpressed in CRC to promote a pro-inflammatory state, characterized by an expansion of tumor-associated macrophages, myeloid-derived suppressor cells, and granulocytic tumor-associated neutrophils (Long et al., 2019). Macrophages of *Enterococcus faecalis*-colonized mice could produce pro-inflammatory cytokine TNF-α, which in turn increased the colon epithelial cell production of netrin-1, a neuronal guidance molecule capable of inhibiting apoptosis to accelerate malignant transformation (Yang et al., 2012).

Obesity is known to produce a chronic, low-grade state of inflammation. Some of the mediator for inflammation are cytokines and inflammation-related molecules (Hursting et al., 2012). A study reported that increased body mass index (BMI) was associated with increase in two proinflammatory colonic cytokines, namely TNF-α and interleukin 6 (IL6), while obesity coincided with precancerous changes in the transcriptome (Pfalzer et al., 2018). Considering the existing correlation between obesity and gut microbiota changes (Boulangé et al., 2016), finding from Pfalzer et al. (2018) implies that inflammation may be one of the mechanisms of how obesity-associated dysbiosis contributes to increased CRC risk.

Although the above describe mainly the detrimental effects of microbiome, *Parabacteroides distasonis*, a commensal organism, has been shown to exert an anti-inflammatory effect. When fed to BALB/C mice, *P. distasonis* could reduce the severity of intestinal inflammation of acute and chronic colitis (Kverka et al., 2011). Likewise, Koh et al. (2020) has reported the beneficial role of *P. distasonis* in attenuating colonic tumorigenesis and maintaining intestinal epithelial barrier in azoxymethane (AOM)-treated mice (Koh et al., 2020).

### Bacterial Toxin

CRC-associated gut microbiota also exerts tumorigenic effect through the production of genotoxin, which induces DNA or chromosomal damage to facilitate malignant transformation. Interestingly, the production of genotoxin is not restricted to only the typically pathogenic bacteria but also commensal bacteria. *E. coli* constitutes a normal component of the gut microbiome, but certain strains that acquire pathogen-like features can produce cyclomodulin genotoxin including colibactin, cytotoxic necrotizing factor (CNF), and cytolethal distending toxin (CDT) (Kurnick et al., 2019), all of which have been examined for their promoting effect in CRC. The pathogenicity island encoding colibactin, known as polyketide synthase (pks), are detected in up to 66.7% of CRC patients (Arthur et al., 2012). Moreover, experimental studies show that colibactin could sustain tumor growth by generating DNA cross links *in cellulo*, which were later transformed into DNA double-strand breaks (Bossuet-Greif et al., 2018; Kawanishi et al., 2019; Wilson et al., 2019), or by favoring the emergence of senescent cells (Cougnoux et al., 2014; Dalmasso et al., 2014). Senescent cells are cells with an irreversible cell cycle arrest. Although early-stage senescence usually protects cells against malignant transformation, a long-term senescence stage changes cellular microenvironment leading to cancer development (Zeng et al., 2018). In the case of CRC, colibactin-induced cellular senescence could lead to the continuous secretion of growth factors that promote tumor growth (Cougnoux et al., 2014; Dalmasso et al., 2014). On the other hand, CNF-encoding gene was found significantly more prevalent in the resection specimens of CRC patients than diverticulosis patients, suggesting the specific role of CNF in promoting colon cancer (Buc et al., 2013). Like colibactin, CNF could induce cellular senescence in human colon cells. Of note, such senescence was reversible, where the polyploid cells re-entered cell cycle, depolyploidised and eventually produced more aneuploid progeny. Given aneuploidy is the hallmark of solid tumor development, the reversible senescence induced by CNF is relevant to colon tumorigenesis (Zhang et al., 2018b). Likewise, CDT that are produced by pathogenic *E. coli* could drive colon carcinogenesis in genetically altered premalignant human colon epithelial cells (Graillot et al., 2016). Another gram-negative bacterium, *Salmonella enterica*, also produces CDT, which has been classified as typhoid toxin due to being a unique virulence factor of *S. enterica* subspecies *enterica* serotype Typhi (Miller and Wiedmann, 2016). Typhoid toxin of *S. enterica* was found to synergize with the loss of the adenomatous polyposis coli (APC) gene to promote microenvironment conducive for malignant transformation, purportedly through the inhibition of DNA repair and DNA damage‐induced cell cycle arrest (Martin et al., 2019a).

Besides *E. coli*, other types of CRC-associated gut microbiota also produce CRC-promoting cytotoxins. The gene encoding *B. fragilis* toxin was detected in the colonic mucosa of both the early-stages and late-stages CRC patients, with a 100% prevalence in the latter (Boleij et al., 2015). Studies investigating the tumorigenicity of *B. fragilis* toxin reported its capability to disrupt or cleave E-cadherin, leading to barrier permeability dysfunction, activation of Wnt/βcatenin and NF-κB signaling pathways as well as expression of IL-8 and IL-17, which altogether form a carcinogenic inflammatory cascade (Wu et al., 2007; Hwang et al., 2013; Chung et al., 2018). *B. fragilis* toxin could also upregulate spermine oxidase, a polyamine catabolic enzyme and subsequently led to the generation of ROS and DNA damage (Goodwin et al., 2011). Although the overall *H. pylori* seroprevalence was not associated with CRC risk, seropositivity to a specific *H. pylori* protein called VacA significantly increased the odds of developing CRC (Epplein et al., 2013; Butt et al., 2019). It was posited that VacA cytotoxin may contribute to CRC by disrupting the ionic equilibrium in enterocytes, primarily through the modulation of chloride concentration in the cellular microenvironment (Ponzetto and Figura, 2019). This mechanism, however, remains as a postulation; the effect of *H. pylori* on CRC is far from clear as previous studies only assessed the antibody responses against VacA in serum samples but did not confirm the presence of *H. pylori* or its associated protein in colorectal tissue samples (Epplein et al., 2013; Butt et al., 2019).

### Bacterial Adherence Factor

Throughout their phylogenetic evolution, bacteria gradually attained virulence factors such as adhesins, pili, and flagella to develop their ability to breach the gut mucosal barrier, as well as to adhere to and to invade intestinal epithelial cells (Gagnière et al., 2016). Studies have elucidated the mechanism to which *F. nucleatum* utilizes its adhesins to bind to and invade host cells: Fusobacterium adhesin A (FadA) binds and shifts away the vascular endothelial cadherin at the endothelial cell-cell junctions to enable the passage of *F. nucleatum* through the loosened junctions of endothelium (Fardini et al., 2011), whereas fibroblast activation protein 2 (Fap2) binds to the D-galactose-β (1-3)-N-acetyl-D-galactosamine (Gal-GalNAc) carbohydrate moiety, which is overexpressed in CRC host, to promote *F. nucleatum* enrichment (Abed et al., 2016). Of note, experimental evidence has indicated that the interaction between bacterial adhesins and their receptors on host cells does not only facilitate bacterial attachment, but also its subsequent pathogenic activities *via* the modulation of different mechanisms. FadA could bind and interact with E-cadherin to induce β-catenin signaling, pro-inflammatory cytokines, and CRC tumor growth in xenograft mice (Rubinstein et al., 2013). A higher expression of FadA was detected in patients with CRC and precancerous adenomas in comparison with non-CRC individuals, further corroborated the facilitative role of FadA in *F. nucleatum*-associated colon carcinogenesis (Rubinstein et al., 2013; Kashani et al., 2020). Similarly, the binding of Fap2 to the human inhibitory receptor T-cell immunoreceptor with Ig and tyrosine based inhibitory motif domains (TIGIT) potentially sustain tumor growth by reducing natural killer cell cytotoxicity and tumor infiltrating lymphocyte cell activities (Gur et al., 2015).

*E. coli*, another CRC-associated gut microbe, also has its adherence and disease-promoting effect mediated by its afimbrial adhesin (afa). Diffusely-adhering *E. coli* expressing afa demonstrates the potential as a pathobiont that promotes IBD or intestinal cancer progression due to its ability to induce intestinal lesions, pro-inflammatory responses, and angiogenesis (Servin, 2014). A study has shown afa-possessing *E. coli* confer better adherence and invasion of intestinal epithelial cells than the afa-negative clones. Moreover, afa had the ability to upregulate the expression of VEGF, which could be linked to angiogenesis and tumor development (Prorok-Hamon et al., 2014). The prevalence of afa-possessing *E. coli* was also found to be higher in CRC as compared to healthy patients, indicating the potential role of afa in mediating the pathogenesis of *E. coli* (Prorok-Hamon et al., 2014; Eklof et al., 2017).

The role of pil3 pilus is essential to the attachment of *S. gallolyticus* to human mucus-producing cells or to the colonization of murine colon (Martins et al., 2015). Interestingly, pil3 bound equally well to the human mucins MUC2 which predominates in healthy colon, and to MUC5AC which is overexpressed only in cancerous colon. It was posited that the ability of *S. gallolyticus* to bind MUC2 mucin *via* pil3 facilitates commensal colonization, while binding to MUC5AC confers a growth advantage over other colon microbiota species in the tumor microenvironment. This helps explain the higher carriage rate of *S. gallolyticus* in the presence of colon tumors (Martins et al., 2016).

### Oxidative Stress and DNA Repair Defects

As discussed above, dysbiotic microbiota provokes chronic gut inflammation *via* different mechanisms. These chronic inflammatory cells may stimulate the release of endogenous ROS and nitrogen species (RONs), which are responsible for the accumulation of different types of DNA damage including single and double strand DNA breaks, DNA crosslinks, thymine glycol, and abasic sites (Ray and Kidane, 2016). One of the gut microbiotas that is capable of triggering such detrimental inflammatory cascade is *E. faecalis*. *E. faecalis* could produce ROS such as extracellular superoxide, which induced tumor-associated chromosomal instability, anaphase bridging, and multipolar mitosis in human colonic epithelial cells (Wang and Huycke, 2007; Wang et al., 2008). *P. anaerobius* interacts with toll-like receptor (TLR) 2 and 4 on colon cells to stimulate an increased ROS level, rate of cholesterol biosynthesis, and colon cell proliferation (Tsoi et al., 2017). Of note, the effect of ROS in carcinogenesis is dichotomous; while it contributes to tumor growth and survival, it induces the apoptosis of cancer cells when present at excessively high level (Lin et al., 2018). There is increasing evidence that ROS involvement in various pathways can lead to the natural resolution of inflammation (Chelombitko, 2018). Hence, the microbiota-induced tumorigenic effect observed in the aforementioned studies could be interpreted as a consequence of oxidative stress, which occurs with prolonged ROS overproduction that could not be compensated by antioxidant systems, rather than the direct tumor promoting effect of ROS itself.

**Figure 2:**
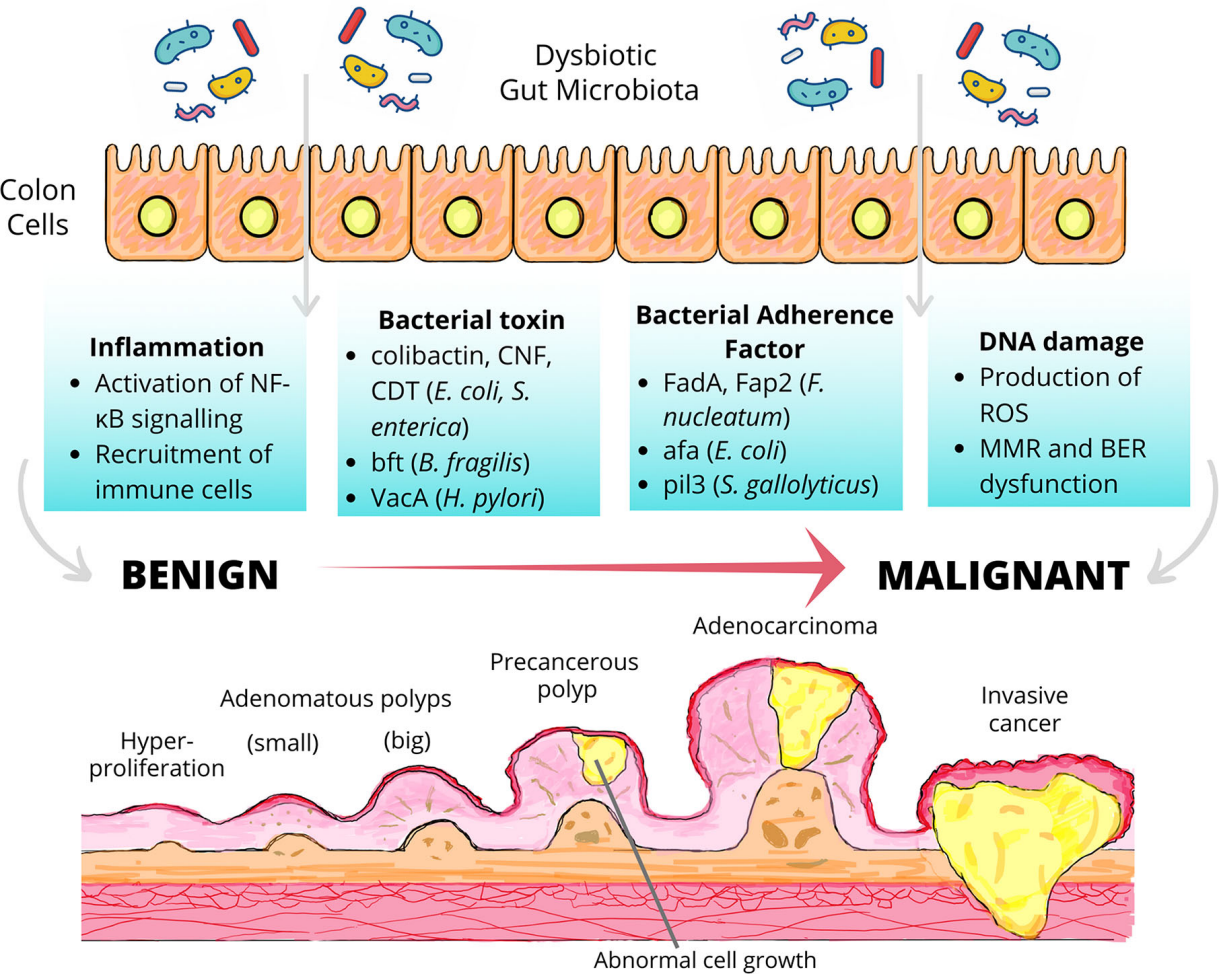
Dysbiosis and colon carcinogenesis. Dysbiotic gut microbiota may drive the malignant transformation of colon cells *via* the induction of inflammation, the secretion of bacterial toxin, the action of bacterial adherence factors and the induction of DNA damage. The transformation of early neoplastic lesions (adenomatous polyps) to colorectal cancer (CRC) may take up to 15 years depending on the characteristics of the lesions and other risk factors including body weight, gender, physical inactivity, etc. (De Palma et al., 2019). NF-κB, nuclear factor-κB; CNF, cytotoxic necrotizing factor; CDT, cytolethal distending toxin; bft, *Bacteroides fragilis* toxin; VacA, vacuolating toxin A; FadA, Fusobacterium adhesin A; Fap2, fibroblast activation protein 2; afa, afimbrial adhesin; pil3, pilus 3; ROS, reactive oxygen species; MMR, mismatch repair system, BER, base excision repair system.

The DNA damage caused by ROS could be repaired by several DNA repair mechanisms including the DNA mismatch repair system (MMR) and base excision repair system (BER), both of which are highly conserved from bacteria to humans (Bridge et al., 2014). The disruption of either of these antioxidant mechanisms by dysbiotic microbiota has been shown to promote carcinogenesis by the accumulation of oxidative stress, which is responsible for the severe cellular and tissue damage as well as chronic inflammation (Chelombitko, 2018). Studies have uncovered the detrimental effect of enteropathogenic *E. coli* (EPEC), which has been shown to significantly reduce the expression of two key MMR proteins, namely MSH2 and MLH1 *via* a post-translation mechanism involving the EPEC effector protein EspF. The MMR dysfunction could also induce microsatellite instability, a phenomenon characterized by the accumulation of DNA replication errors particularly in the area of short repetitive DNA stretches (Maddocks et al., 2009; Maddocks et al., 2013). Considering the fact that microsatellite instability is detected in up to 20% of CRC (Nojadeh et al., 2018), investigations into how other gut microbiota cause MMR dysfunction become a matter of great clinical importance. Besides, it has been proposed that dysbiotic microbiota may promote the accumulation of carcinogenic BER intermediates, primarily AP sites, leading to genomic instability and colon carcinogenesis (Ray and Kidane, 2016). Moreover, the polymorphism of BER genes could modulate CRC risk, presumably, by influencing the BER processes (Brevik et al., 2010; Kabzinski et al., 2016). Nonetheless, whether or not specific gut microbiota could interfere with BER, causing its loss of biological function and subsequently influence risk of colon carcinogenesis is yet to be explored. **Figure 2** summarizes the mechanisms to which dysbiosis may set the stage for colon carcinogenesis.

## Possible Preventive and Therapeutic Strategy for Colorectal Cancer: the Probiotics

Probiotics are typically defined as living microorganisms, which when administered in adequate amounts, confer health benefits to the host. The potential contribution of probiotics to the preventative or therapeutic strategies for CRC is attributed to its ability to first, inhibit colonization by pathogenic bacteria; second, to modulate the gut immunity; and third, to strengthen the gut barrier (Fong et al., 2020). The inhibitory effect of probiotics against pathogenic bacteria has been demonstrated by several studies. The consumption of *Bacillus* bacteria was shown to inhibit intestinal colonization by *Staphylococcus aureus via* the interference of its quorum sensing signaling (Piewngam et al., 2018). *Clostridium butyricum* exhibited inhibitive effect against biofilm formation by enterotoxigenic *B. fragilis*, *via* the regulation of several *B. fragilis* virulence and efflux pump-related genes (Shi et al., 2020). Specific *Lactobacillus* strains, namely *Lactobacillus fermentum* 88 and *Lactobacillus plantarum* 9, demonstrated high adhesion values to the human enteric cell line HT29 while inhibiting the adhesion of *E. coli* to HT29 (Gharbi et al., 2019). Pre-treatment with *Lactobacillus rhamnosus* GG prior to the experimental induction of periodontitis in mouse model also exerted protective effect against *F. nucleatum* and *Porphyromonas gingivalis*-induced caecum dysbiosis, as well as significantly reduced intestinal inflammation (Gatej et al., 2020).

As mentioned, probiotics play an immunomodulatory role in the gut and can reduce colonic inflammation. Through the suppression of Wnt/β‑catenin signaling, *Lactobacillus* species was shown to ameliorate colonic inflammation and tumor growth in azoxymethane (AOM) and dextran sulfate sodium (DSS)-induced CRC murine model (Ghanavati et al., 2020). Treatment with a specific strain of *L. rhamnosus* decreased tumor incidence by inhibiting inflammation and promoting apoptosis (Gamallat et al., 2016). *In* APC*Min/+* mouse model of colon cancer, *L. plantarum* strain YYC-3 prevented colon tumor development, putatively by suppressing the production of inflammatory cytokines and infiltration of inflammatory cells (Yue et al., 2020). Administration of a mixture of *Lactobacillus acidophilus*, *L. rhamnosus* and *Bifidobacterium bifidum* decreased colitis and resulted in a 40% lower number of tumors than the control group (Mendes et al., 2018).

**Figure 3:**
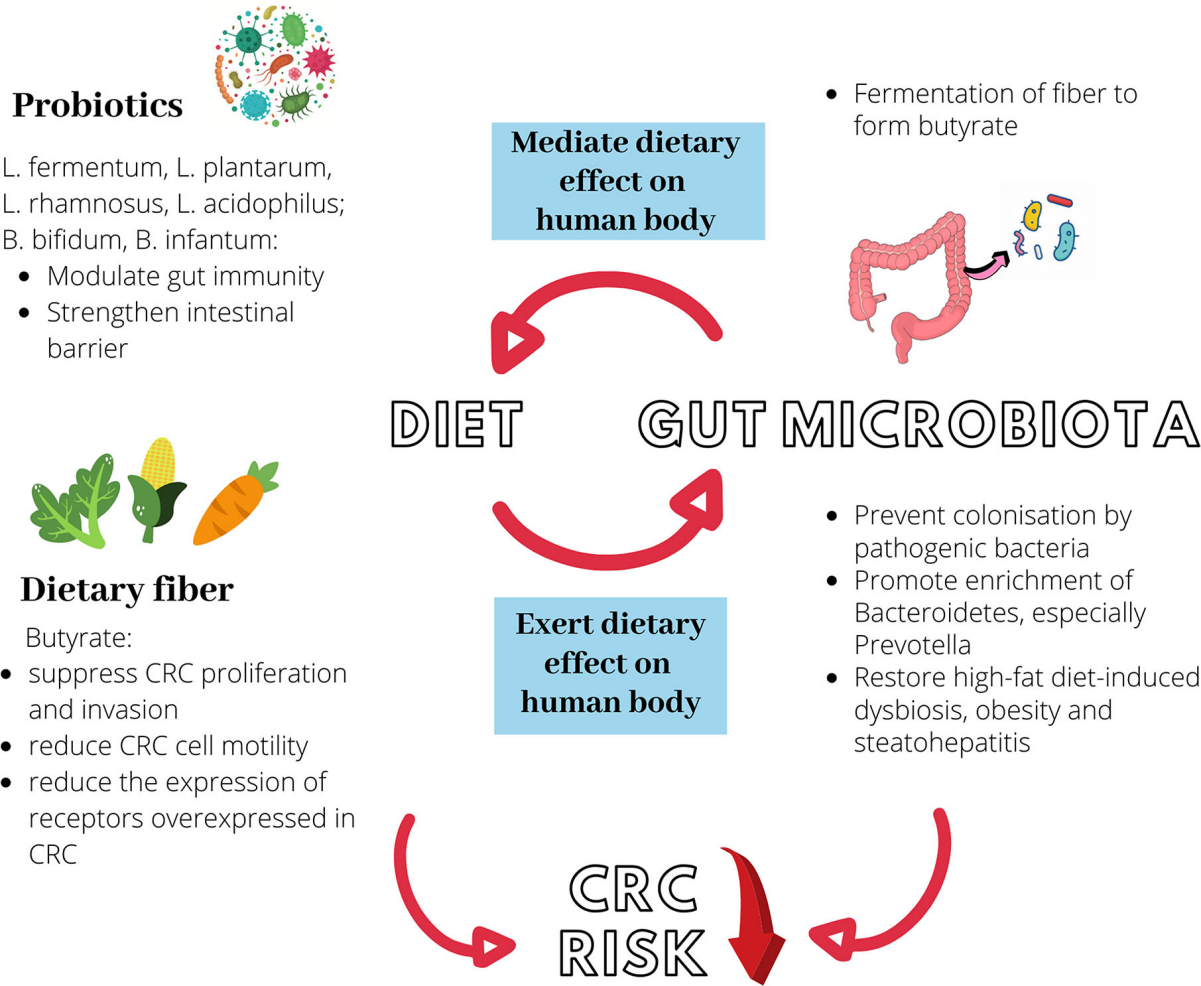
Diet, gut microbiota, and decreased colorectal cancer (CRC) risk. The fermentation of dietary fiber by gut microbiota results in the production of butyrate. Butyrate exerts inhibitory effect against colon carcinogenesis through several mechanisms, including the restoration of gut dysbiosis induced by high-fat diet. Probiotics such as *Lactobacillus* and *Bifidobacterium* prevents gut colonization by pathogenic bacteria, modulates gut immunity, and maintains gut barrier integrity.

It is known that a dysregulation of tight junction proteins including occludin, claudins, junctional adhesion molecules, and zona occludens (ZO) can lead to a leaky epithelial barrier, setting the stage for intestinal inflammation and IBD-associated CRC (Landy et al., 2016). The ability of probiotics to enhance gut barrier integrity can hence play a preventive role. A study previously reported that the probiotic *E. coli* Nissle 1917 could enhance intestinal barrier function *via* the upregulation and redistribution of the tight junction proteins ZO-1, ZO-2, and claudin-14 (Alvarez et al., 2016). A probiotic mixture comprising *Bifidobacterium*, *L. acidophilus*, and *Enterococcus* attenuated colitis in mice by upregulating the expression levels of occludin and claudin-4 (Zhang et al., 2018a). In 1,2-dimethylhydrazine dihydrochloride (DMH)-induced CRC mouse model, administration of *L. acidophilus*, *B. bifidum*, and *Bifidobacterium infantum* enhanced TLR2 signaling and gut mucosa epithelial barrier integrity, both of which correlated with decreased tumor incidence (Kuugbee et al., 2016). **Figure 3** illustrates the interactions between diet, gut microbiota, and decreased risk of CRC.

## Conclusion And FUture Directions

This report has examined recent scientific findings that highlighted the active participation of gut microbiota in the pathogenesis of CRC *via* different types of mechanisms. The purported mechanisms, however, do not indicate whether the state of the gut microbiota is a cause or a consequence of colon carcinogenesis. CRC-associated microbiota have long been grouped into driver species comprising pro-carcinogenic gut bacteria that initiate CRC development, or passenger species comprising opportunistic pathogens that outcompete the driver species in the tumor microenvironment (Tjalsma et al., 2012). In that sense, driver species can be regarded as the initiator of colon carcinogenesis while the passenger species is the consequence which further aggravates tumor progression after gaining colonization advantage in the established tumor microenvironment. Several recent studies however, redefine the roles of CRC-associated microbiota differently from what was originally proposed: *E. coli*, initially thought to be a driver species, may be a passenger species capable of replacing other gut microbiota through the action of colibactin (Wassenaar, 2018); *S. gallolyticus*, initially thought to be a passenger species, may be a driver species capable of inducing colon carcinogenesis on its own (Pasquereau-Kotula et al., 2018). Indeed, the existing scientific evidence is insufficient to provide a definitive answer for the cause-or-effect debate revolving around the role of gut microbiota in CRC. With more robust studies that could decipher the mechanisms underlying the pathogenicity of CRC-associated gut microbiota, the causative role of particular gut microbiota may one day be established, analogous to how *H. pylori* has been proven to be a strong risk factor for the development of gastric cancer (Wroblewski et al., 2010). Investigations into the exact effect of microbial dysbiosis on CRC development is a subject of tremendous clinical importance as gut microbiota is postulated to contribute to CRC progression and maintenance, as well as to define patients’ prognosis (Mima et al., 2016; Wei et al., 2016; Chen et al., 2019) and response to anti-cancer therapies (Pouncey et al., 2018; Lin et al., 2019; Villéger et al., 2019).

Evidence from epidemiological, animal, and human cell line studies also strongly support the influence of dietary factors on CRC risk. The findings so far converge to indicate that CRC risk could be reduced by an increased intake of dietary fiber while an elevated CRC risk is associated with the excessive intake of red and processed meat, saturated fats and to a smaller extent, simple sugar. These dietary components exert their detrimental effect by altering the composition and diversity of the gut microbial community to increase dysbiosis-associated CRC risk. On the other hand, dietary impact on the human body is itself mediated by the gut microbiota activity, which means dysbiosis triggered by any factor can add to CRC risk by shifting the effect of dietary components toward promoting colonic neoplasm. In either direction of this two-way interaction, dietary components mediate the risk of malignant transformation in the colon by interacting with gut microbiota. Of note, the evidence of dietary impacts on gut microbiota obtained from animal studies must be interpreted with caution as the gut microbiota of in-bred animal may not reflect the real-life gut microbiota of humans, which is shaped by many factors other than diet (Nguyen et al., 2015). Additionally, many human studies linking dietary habits, gut microbiota and CRC risk are cross-sectional. There is a pressing need to establish more well-designed longitudinal studies that assess the gut microbiota composition, dietary habits and gastrointestinal health status across different life stages, in order to determine the causal effects of dysbiosis and dietary compounds on CRC development and progression. It would also be of interest to determine if a stool-based screening of microbial dysbiosis can be utilized as a non-invasive and affordable CRC screening tool, which is eminently useful in resource-poor regions where colonoscopy is not readily available. Certainly, a standardized protocol with higher specificity and sensitivity must be developed before such screening tool can be implemented.

Probiotics have long been posited as a prophylactic and even therapeutic measure in colon carcinogenesis. While probiotics have demonstrated great health benefits, it should be noted that probiotics use is accompanied by risk; host response toward gut microbiome intervention varies from individual to individual, with likelihood of adverse effect among individuals with impaired gut barrier and compromised immunity (Doron and Snydman, 2015). More studies are warranted to confirm the risk and benefits associated with probiotics use in individuals with underlying medical conditions, to ensure that only probiotics with definitive protective effects against CRC are integrated into the clinical management of CRC. A personalized microbiome therapy taking into account the host genetics, physiology, and immunity will contribute to a higher success rate of such intervention.

## Author Contributions

YL and MC designed the study and wrote the manuscript. YN, WL, and SP reviewed and revised the manuscript. MC and WL acquired the funding. All authors contributed to the article and approved the submitted version.

## Funding

This article is funded by the matched fund of Sunway Medical Centre and Sunway University: GRTEX-OTR-CBP-SMC-001-2019.

## Conflict of Interest

The authors declare that the research was conducted in the absence of any commercial or financial relationships that could be construed as a potential conflict of interest.
